# Acute Anterior Uveitis with HLA-B27 Positivity after Corneal Cross-Linking with Previous Intrastromal Corneal Ring Segment

**DOI:** 10.1155/2022/2014549

**Published:** 2022-11-21

**Authors:** Asher Khan, Charles D. McGuffey, Andrew T. Melson, Kamran M. Riaz

**Affiliations:** ^1^Dean McGee Eye Institute, 608 Stanton L. Young Blvd, Oklahoma City, OK 73104, USA; ^2^College of Medicine, University of Oklahoma, 800 Stanton L. Young Blvd, Oklahoma City, OK 73117, USA

## Abstract

*Purpose*. To describe a case of acute anterior uveitis (AAU) with HLA-B27 positivity following epithelium-off corneal cross-linking (CXL) in a patient with a previous intrastromal corneal ring segment. *Observations.* A 28-year-old male with keratoconus (KCN) developed ophthalmalgia, perilimbal injection, hypopyon, and decline in corrected distance visual acuity (CDVA) 3 days after CXL. A working diagnosis of inflammatory versus infectious AAU was made, and the patient was treated with topical tobramycin, polymyxin B/trimethoprim, prednisolone, and oral valacyclovir. Clinical appearance and CDVA improved, ultimately returning to baseline by two weeks postoperatively. Diagnostic laboratory workup revealed HLA-B27 positivity. *Conclusions and Importance.* A comprehensive laboratory workup is helpful to identify potential causative and associated systemic conditions when encountering AAU after CXL. Given the overlap in patient demographics for KCN and HLA-B27 positivity, clinicians should consider this entity in the differential diagnosis and treatment of such cases.

## 1. Introduction

Acute anterior uveitis (AAU) is a type of intraocular inflammation of the iris and/or ciliary body, defined by the presence of cells or cellular aggregates in the anterior chamber. AAU may be primary or secondary to a variety of etiologies, including infectious, noninfectious, and masquerade diseases. AAU has a well-recognized association with HLA-B27, an antigen located on the surface of white blood cells, especially in young- to middle-aged males [1].

Several treatment options exist for the treatment of mild-moderate keratoconus (KCN). Intrastromal corneal ring segments (ICRS) are small, crescent-shaped polymethylmethacrylate rings placed in the deep, peripheral corneal stroma to flatten the cornea and decrease refractive error in ectatic corneas [2]. Corneal cross-linking (CXL) involves treatment of the cornea with riboflavin followed by photoactivation with UVA light, leading to corneal strengthening. CXL can be performed with or without removing the corneal epithelium. The former technique is well-documented in the literature as a protocol to halt the progression of KCN and is the only technique approved for on-label CXL use in the United States at this time [3]. While a number of noninfectious complications can occur after CXL, post-CXL AAU has been reported only twice before in the literature: once in association with X-linked chronic granulomatous disease (CGD) and once following a combined CXL/ICRS procedure [4, 5].

We present a case of post-CXL AAU in a KCN patient with preexisting ICRS wherein laboratory testing revealed HLA-B27 positivity without previous diagnosis or systemic pathology. Although our patient's condition resolved with treatment, we bring special attention to post-CXL AAU as a clinical finding that merits comprehensive laboratory testing for appropriate diagnosis and long-term management.

## 2. Case Report

A 28-year-old white male with moderate KCN had previously undergone epithelium-off CXL of the right eye approximately 8 years prior with an unremarkable postoperative course. At our institution, the patient underwent ICRS placement (Intacs-Addition Technology, Inc., Sunnyvale, CA, USA) in the left eye 8 months prior with an unremarkable postoperative course. Besides KCN, the patient had no significant ocular history, ocular surgery history, medical history, family history, social history, or medication use. The patient's last known refraction in the left eye prior to CXL was −11.50 + 0.75 × 15 with a corrected distance visual acuity (CDVA) of 20/40. A diagnosis of progressive KCN was made based on serial tomography measurements demonstrating approximately 1.8 D worsening of maximum keratometry (KMax) over the past eight months. The ultrasonic pachymetry at this time was 459 *μ*m.

The patient subsequently underwent epithelium-off (Dresden protocol) CXL in the left eye (OS). Postoperatively, a bandage contact lens (BCL; AirOptix Night and Day, BC 8.4, diameter 13.8; Alcon Laboratories, Fort Worth, TX, USA) was placed, and the patient was instructed to use topical moxifloxacin 4 times daily with postoperative follow-up five days later. Three days later (two days before the scheduled follow-up visit), the patient presented with significant ophthalmalgia, perilimbal injection, hypopyon, and a decline in corrected distance visual acuity (CDVA).

**Figure 1:**
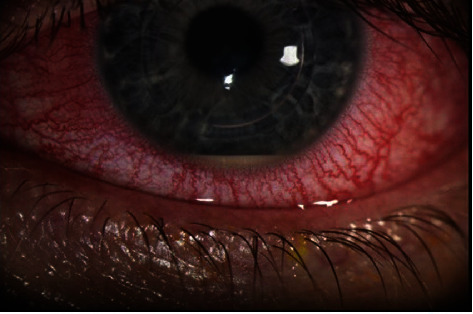
Slit beam photograph of the left eye. This photograph demonstrates 2+ perilimbal injection and 1-1.5 mm inferior hypopyon observed at the time of initial presentation. There is no overlying epithelial defect or appreciable infiltrate in the cornea. Both intrastromal corneal ring segments are in place.

At presentation, CDVA was 20/1000 OS. Pupils were equally reactive in both eyes with no relative afferent pupillary defect. Intraocular pressure measured 6 mmHg and ultrasound pachymetry measured 401 *μ*m OS. Slit-lamp exam of the left eye revealed 2+ perilimbal injection and a well-placed ICRS without extrusion or epithelial defect. Notably, there was no corneal infiltrate appreciated on exam. Anterior chamber exam was notable for 2+ cells and a 1-1.5 mm inferior hypopyon (Figure 1). Anterior segment exam of the right eye and dilated fundus exam of both eyes were without evidence of intraocular inflammation.

**Figure 2:**
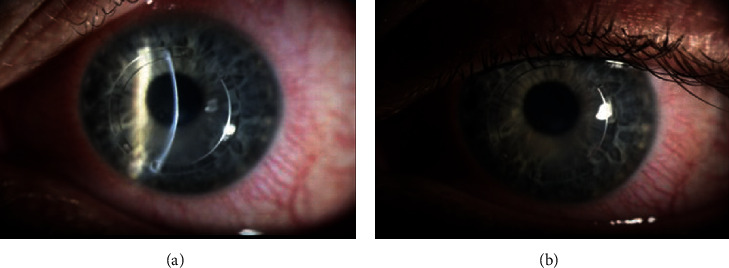
Slit beam (a) and broad beam (b) photographs of the left eye. These photographs demonstrate regression of the perilimbal injection and inferior hypopyon observed in Figure 1.

**Figure 3:**
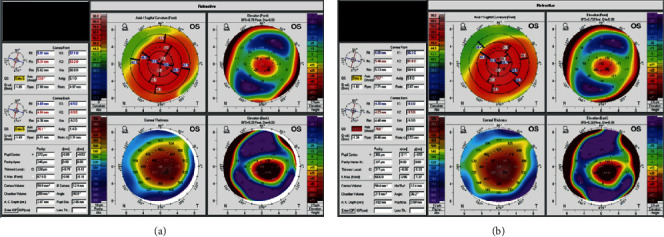
Tomography images of the left eye before (a) and after (b) cross-linking at the last follow-up 6 months postoperatively demonstrate the stability of keratoconus over the time course from before and after surgery.

The patient denied any previous history of intraocular inflammation, infectious keratitis, or receiving any vaccine (including recent influenza or COVID-19 vaccination) within the month leading up to symptoms but did endorse a remote history of occasional cold sores around the mouth and nose. Anterior chamber paracentesis was offered at this time, but the patient declined this invasive procedure. A working diagnosis of inflammatory versus infectious AAU was made, and the patient was started on topical tobramycin 0.3%, polymyxin B/trimethoprim, prednisolone acetate 1%, and oral valacyclovir 1 gram, all to be taken three times daily. As there were no overt signs of corneal infection such as corneal ulcer, epithelial defect, or infiltrate, corneal cultures were not obtained with consideration to avoid violation of the corneal epithelium. The patient tolerated this medication regimen without any adverse effects. Three days later, CDVA improved to 20/60, and regression of the hypopyon and perilimbal injection was noted (Figure 2). Tobramycin was discontinued, polymyxin B/trimethoprim and valacyclovir were continued, and prednisolone was increased to 6 times daily. At postoperative week 2, intraocular pressure measured 6 mmHg OS, and the ophthalmalgia, hypopyon, and perilimbal injection had completely resolved. Consequently, topical antibiotics were stopped, a prednisolone taper was initiated, and valacyclovir was reduced to once every other day dosing. All medications were stopped by three months postoperatively. No recurrences of AAU were noted over the next 6 months. Tomography imaging of the left eye revealed stability of KCN before and after CXL at the last follow-up (Figure 3).

Due to the unexpected AAU after CXL, the patient underwent comprehensive laboratory testing (Appendix) on the postoperative day three visit. All were normal besides HLA-B27, which was positive. Notably, the patient denied any previous history of extraocular inflammatory symptoms commonly associated with HLA-B27 positivity, including low back pain, arthritis, urethritis, and gastrointestinal changes [6]. The patient was educated on the systemic significance of the diagnosis, informed about the need for continued KCN monitoring, and subsequently referred to his primary care physician and a rheumatologist for continuity of care.

## 3. Discussion

While AAU can be secondary to several different etiologies, it has a well-established association with HLA-B27, especially in young white males [1]. Inclusive of all causes, AAU remains a relatively rare event after CXL. A previous study found that out of 153 eyes that underwent epithelium-off CXL, only 1.9% developed AAU during the first 3 postoperative months, though none of the patients had an ocular or systemic etiology typically associated with uveitis [7].

The use of topical steroids after CXL is a matter of debate. While many surgeons routinely employ topical steroids to control postoperative inflammation, a recent systematic review reported that a combination of steroids and bandage contact lenses postoperatively is associated with infectious keratitis after CXL [8]. The treating surgeon's (KMR) protocol is to only use topical antibiotics after CXL with BCL placement, and patients start topical steroids on the postoperative day 5-7 visit, after BCL removal and confirmation of epithelialization. It is plausible that the lack of topical steroids immediately after CXL contributed significantly to the development of AAU.

HLA-B27-associated AAU typically affects young males and may be associated with extraocular inflammatory disease [1, 9]. Our patient's AAU presentation was characteristic since there was an acute onset of blurred vision, ocular redness, pain, and hypopyon. This last finding is significant as hypopyon rarely develops in the context of noninfectious uveitic etiologies with a notable exception of HLA-B27, Behcet's disease, or drug use [10]. Furthermore, the patient's symptoms were unilateral and lasted for a limited duration, both additional typical characteristics of HLA-B27-associated AAU [11]. Treatment strategies for HLA-B27-associated AAU most frequently involve topical corticosteroids, with or without topical anticholinergic agents, which are slowly tapered over 8-10 weeks [1, 9, 11].

Several lessons may be of interest from our case. First, our case adds unique knowledge to the literature as it reports HLA-B27 positivity in laboratory workup for AAU after CXL in a patient without prior systemic disease and with preexisting ICRS. Our search protocol consisted of a PubMed search without any filters, using the search terms “uveitis”, “crosslinking”, “HLA-B27”, and “keratoconus.” Second, we note that for patients presenting with unexpected intraocular inflammation after CXL, a thorough laboratory workup may be of significant utility in identifying common etiologies of AAU, including HLA-B27 positivity. We emphasize that such cases should not be presumed as idiopathic. Diagnostic testing, while seemingly cumbersome and expensive, can assist clinicians to identify associated etiology and prompt appropriate systemic evaluation. A multidisciplinary approach may be needed to manage both ocular and systemic pathology in such cases, especially if the causative etiology is known to cause significant morbidity.

Previous authors have reported AAU following CXL, but in one report, the AAU was thought to be attributable to X-linked CGD [4]. In the other report, the patient developed AAU after a simultaneous CXL/ICRS procedure, thought to be due to a combination of the patient self-replacing the BCL, sterile corneal epithelial defect, and inflammation secondary to free radical formation by riboflavin [5]. The authors did not report any systemic causes or positive diagnostic testing and attributed the anterior chamber inflammation to be similar to sterile anterior chamber reaction after laser refractive surgery. In our case, since there was no known history of systemic autoimmune disease and the ICRS was preexisting, we suspect that the postsurgical inflammatory state exacerbated a relatively dormant hyperimmune state that led to the AAU episode. While CXL is not an intraocular procedure, there are numerous aspects of the procedure that may incite ocular inflammation, such as potential free radical damage by riboflavin and mechanical damage from epithelial removal upregulating ocular surface inflammation [12].

Although our patient underwent CXL of the right eye approximately 8 years prior at a different institution, there were no postoperative uveitic changes observed at that time. Since the CXL in both eyes was performed using the same protocol (Dresden protocol), it is unlikely that differences between the surgeries, such as different institutions or surgeons, account for the dissimilarity in postoperative courses. Instead, one variable that changed between the surgeries and may help explain the different outcomes is the patient's age. Other authors have reported the average age of onset for HLA-B27-positive ankylosing spondylitis (AS) in males as 25.7 years [13]. Since our patient was 20 years old at the time of the first CXL and 28 years old at the time of the second CXL, we postulate that the patient may have been more prone to developing uveitic changes associated with HLA-B27 positivity. However, we acknowledge that this is a tenuous explanation at best, and it remains unclear as to why AAU did not develop in the first eye after CXL. Furthermore, given the lack of extraocular and systemic symptoms, HLA-B27 positivity may not be the sole etiology for post-CXL AAU. Nevertheless, our case sheds light on how the appearance of post-CXL AAU led us to perform diagnostic testing which confirmed HLA-B27 positivity. Ultimately, the diagnostic implications are of the most significance as they relate to patients' long-term ocular and systemic well-being.

Other possible risk factors include the use of a BCL postoperatively. Despite the relatively steep base curve of the BCL used, it may have been too loose (flat) for this patient's KCN cornea, further causing mechanical irritation to the denuded corneal surface. Additionally, corneal trauma sustained from the CXL procedure itself may have led to the inflammation seen in our patient, as the irradiance delivered could have surpassed the endothelial damage threshold [5, 12, 14].

In both cases of AAU following CXL in the existing literature, corneal specimens were sent for gram stain, bacterial culture, and herpes simplex virus polymerase chain reaction, with all tests yielding negative results in both patients [4, 5]. Other authors have reported that herpetic keratitis reactivation can occur after CXL, and given our patient's remote history of cold sores, we chose to treat with oral valacyclovir empirically [15, 16]. As there were no corneal exam findings to suggest an infectious etiology, corneal scraping and/or an anterior chamber tap were deferred. Instead, we based our management on the clinical exam findings and laboratory workup as detailed in the Appendix. Clinicians may want to modify or expand this workup to include invasive testing in recalcitrant cases to rule out atypical or infectious causes of AAU.

Patients diagnosed with HLA-B27-associated AAU carry an almost sevenfold increased relative risk of concomitant AS [9]. Interestingly, the demographic predilection of both KCN and AS involves a similar cohort of patients, namely, young males. While previous views held that KCN is primarily a noninflammatory disease, there is increasing evidence to confirm the role of ocular and systemic inflammation in its pathogenesis and progression [17]. Prior studies emphasize additional overlap between KCN and AS by depicting how AS induces corneal biomechanical changes similar to those seen in KCN, characterized by decreases in corneal thickness, stiffness, and hysteresis [14, 18, 19]. This knowledge may allow providers to consider appropriate coinvolvement of corneal, uveitis, and systemic specialists when clinical and laboratory findings are present.

## 4. Conclusions

AAU is a complication that clinicians should be aware of when performing CXL. A thorough ocular and systemic history should be obtained before CXL. If clinicians observe AAU postoperatively, the intraocular findings should not be simply assumed to be idiopathic or routine postsurgical inflammation. In such cases, a thorough yet tailored laboratory workup should be considered, as it may have implications for the future management of ocular and systemic disease.

## Appendix

Due to the unexpected AAU after CXL, the patient underwent laboratory testing, including complete blood count, complete metabolic panel, ESR, CRP, RPR, FTA-ABS, HLA-B27, IGRA, ACE, lysozyme, HSV IgG/IgM, HZV IgG/IgM, CMV IgG/IgM, EBV IgG/IgM, Lyme IgG/IgM, Toxoplasma IgG/IgM, Toxocariasis IgG/IgM, ANA, RF, ANCA titers, SS-A/SS-B, and HIV-1/2.

## Abbreviations

ESR: Erythrocyte sedimentation rate
CRP: C-reactive protein
RPR: Rapid plasma reagin
FTA-ABS: Fluorescent treponemal antibody absorption
HLA-B27: Human leukocyte antigen B27 (subtypes B^∗^2701-2759)
IGRA: Interferon-gamma release assay
ACE: Angiotensin-converting enzyme
IgG/IgM: Immunoglobulin G/M
HSV: Herpes simplex virus
HZV: Herpes zoster virus
CMV: Cytomegalovirus
EBV: Epstein-Barr virus
ANA: Anti-nuclear antibody
RF: Rheumatoid factor
ANCA: Anti-neutrophil cytoplasmic antibody
SS-A: Sjögren's syndrome-related antigen A
SS-B: Sjögren's syndrome type B antigen
HIV: Human immunodeficiency virus.

## References

[1] Chang J. H., McCluskey P. J., Wakefield D. (2005). Acute anterior uveitis and HLA-B27. *Survey of Ophthalmology*.

[2] Zadnik K., Money S., Lindsley K. (2019). Intrastromal corneal ring segments for treating keratoconus. *Cochrane Database of Systematic Reviews*.

[3] Kolli S., Aslanides I. M. (2010). Safety and efficacy of collagen crosslinking for the treatment of keratoconus. *Expert Opinion on Drug Safety*.

[4] Wisely C. E., Daluvoy M. (2021). Anterior uveitis following collagen crosslinking in a patient with X-linked chronic granulomatous disease. *Canadian Journal of Ophthalmology*.

[5] Goldich Y., Elbaz U., Rootman D. S. (2015). Anterior uveitis after collagen cross-linking for keratoconus. *International Journal of Keratoconus and Ectatic Corneal Diseases*.

[6] Parameswaran P., Lucke M. (2021). *HLA B27 Syndromes*.

[7] Cagil N., Sarac O., Cakmak H. B., Can G., Can E. (2015). Mechanical epithelial removal followed by corneal collagen crosslinking in progressive keratoconus: short-term complications. *Journal of Cataract and Refractive Surgery*.

[8] Murchison C. E., Petroll W. M., Robertson D. M. (2021). Infectious keratitis after corneal crosslinking: systematic review. *Journal of Cataract and Refractive Surgery*.

[9] D'Ambrosio E. M., La Cava M., Tortorella P., Gharbiya M., Campanella M., Iannetti L. (2017). Clinical features and complications of the HLA-B27-associated acute anterior uveitis: a metanalysis. *Seminars in Ophthalmology*.

[10] Zaidi A. A., Ying G. S., Daniel E. (2010). Hypopyon in patients with uveitis. *Ophthalmology*.

[11] Wakefield D., Clarke D., McCluskey P. (2021). Recent developments in HLA B27 anterior uveitis. *Frontiers in Immunology*.

[12] Çakmak S., Sucu M. E., Yildirim Y. (2020). Complications of accelerated corneal collagen cross-linking: review of 2025 eyes. *International Ophthalmology*.

[13] Feldtkeller E., Khan M. A., van der Heijde D., van der Linden S., Braun J. (2003). Age at disease onset and diagnosis delay in HLA-B27 negative vs. positive patients with ankylosing spondylitis. *Rheumatology International*.

[14] Ozarslan Ozcan D., Ozcan S. C., Kimyon G. (2022). Corneal biomechanical parameters in systemic autoimmune diseases. *Clinical & Experimental Optometry*.

[15] Kymionis G. D., Portaliou D. M., Bouzoukis D. I. (2007). Herpetic keratitis with iritis after corneal crosslinking with riboflavin and ultraviolet A for keratoconus. *Journal of Cataract and Refractive Surgery*.

[16] Al-Qarni A., AlHarbi M. (2015). Herpetic keratitis after corneal collagen cross-linking with riboflavin and ultraviolet-A for keratoconus. *Middle East African Journal of Ophthalmology*.

[17] Nemet A. Y., Vinker S., Bahar I., Kaiserman I. (2010). The association of keratoconus with immune disorders. *Cornea*.

[18] Caglayan M., Sarac O., Kosekahya P., Erten S., Ayan B., Cagil N. (2017). Biomechanical evaluation of cornea in patients with ankylosing spondylitis. *International Ophthalmology*.

[19] Fontes B. M., Ambrósio R., Velarde G. C., Nosé W. (2011). Ocular response analyzer measurements in keratoconus with normal central corneal thickness compared with matched normal control eyes. *Journal of Refractive Surgery*.

